# Wheezing after the use of acetaminophen and or ibuprofen for first episode of bronchiolitis or respiratory tract infection

**DOI:** 10.1371/journal.pone.0203770

**Published:** 2018-09-13

**Authors:** Paul Walsh, Stephen J. Rothenberg

**Affiliations:** 1 Pediatric Emergency Medicine, Sutter Medical Center, Sacramento, CA, United States of America; 2 Instituto Nacional de Salud Pública, Centro de Investigación en Salud Poblacional, Cuernavaca, Morelos, Mexico; Universita degli Studi di Ferrara, ITALY

## Abstract

**Background:**

Bronchiolitis sometimes triggers the development of subsequent recurrent wheezing. Treatment with either acetaminophen or ibuprofen during the initial episode may affect the occurrence of subsequent wheezing.

**Materials and methods:**

We did a retrospective study comparing the effect of prescribing acetaminophen, ibuprofen, or neither for a first episode of bronchiolitis on medical attendances for subsequent wheezing in infants younger than 12 months. We created our cohorts using California Medicaid data from 2003 to 2010. We used propensity score derived inverse probability weights to adjust for non-random drug assignment. We used robust negative binomial regression to model incident rate ratios (IRR) for medical attendances at 365, 30, and 14-day follow-up. We did similar analyses for the effect of antipyretics for a first medically attended upper respiratory tract infection (URI) on subsequent wheezing.

**Results:**

Compared with no antipyretic, treatment with acetaminophen or ibuprofen for a first episode of bronchiolitis was associated with decreased wheezing at 365-day follow-up (IRR 0.18, 95% CI 0.15–0.22), and ibuprofen plus acetaminophen over ibuprofen (IRR at 0.12, 95% CI 0.05–0.32). The results were similar at 30 and 14-day follow-up. Ibuprofen alone and ibuprofen plus acetaminophen were associated with decreased visits for subsequent wheezing at 365-day (IRR 0.79, 95% CI 0.68–0.92), but not earlier timepoints, when compared with acetaminophen. A smaller effect was seen for ibuprofen at one year if prescribed for a URI (IRR 0.87, 95% CI 0.76–1.00) but not at earlier follow-up.

**Conclusion:**

Children who are prescribed antipyretics for a first episode of bronchiolitis may have less subsequent wheezing than those who are not. We found fewer visits for subsequent wheezing for those prescribed ibuprofen, and ibuprofen combined with acetaminophen, compared with acetaminophen alone.

## Introduction

Bronchiolitis, particularly when caused by respiratory syncytial virus (RSV), is frequently, followed by recurrent wheezing or asthma. RSV induces the formation of IgE and IgG_4_ to bystander antigens, thereby leading to wheezing when these antigens are encountered subsequently.[1–3] Another potential mechanism involves the child’s interferon-γ and IL-13 responses, which are similar in direction but greater in magnitude for rhinovirus than RSV, particularly in children under 2 years of age.[4] [5]

Non-steroidal anti-inflammatory drugs (NSAIDs) inhibit COX and demonstrate promising immunologic effects that may decrease late wheezing in animal models.[6] Trials in farm animals have generally shown clinical but not histological benefits favoring NSAIDs over placebo in respiratory infections in general and in RSV in particular.[7] [8] [9]Randomized controlled trials (RCT) comparing ibuprofen and acetaminophen in older children with asthma have had mixed results.[10, 11] Epidemiological evidence is also mixed but slightly favors NSAIDs over acetaminophen.[12–15] Potential harms from NSAIDs and acetaminophen include increased viral shedding because of the drugs’ immunomodulating effect.[7, 16, 17]

An RCT to compare acetaminophen and ibuprofen in the treatment of bronchiolitis will face three empiric problems. First, is the question of whether a difference between acetaminophen with ibuprofen reflects more benefit or less harm than using neither drug. Second, if antipyretics do confer a benefit, it is not known how early in the course of bronchiolitis that NSAIDs or acetaminophen must be given to realize a benefit. If antipyretics must be given during the upper respiratory tract (URI) phase of bronchiolitis, then infeasibly large numbers of patients would be required because most URIs will not become bronchiolitis. Finally, any effect is likely modest so that even if treatment can be started later when bronchiolitis is manifest an RCT will need to enroll a lot of infants.

### Objectives

We had three objectives:

Objective 1: To compare the incidence of subsequent wheezing in children treated with ibuprofen, acetaminophen, both, or neither for a first wheezing episode or bronchiolitis. This analysis will estimate the direction of treatment effect of antipyretics.

Objective 2: To simulate to the extent possible a blocked RCT comparing the incidence of subsequent wheezing in children treated with ibuprofen, acetaminophen, or both for URI.

Objective 3: To simulate to the extent possible a blocked RCT comparing the incidence of subsequent wheezing in infants treated with ibuprofen, acetaminophen, or both, for a first wheezing episode or bronchiolitis.

We complete each of these objectives using the terms Cohort 1 through 3 respectively.

## Materials and methods

The State of California Committee for Protection of Human Subjects and the State of California Department of Health Care Services (DHCS) approved this study. The data was masked by a DHCS analyst prior to being released to us. This is not the same as anonymization and because of the potential to re-identify subjects by combining these data with publicly available data, the Committee for Protection of Human Subjects has mandated the destruction of the dataset following completion of the study. The Committee for Protection of Human Subjects (State institutional review and privacy board) has waived the requirement to obtain written informed consent from each patient, parent, or guardian.

### Data source

The DHCS is the primary health care payer for low income children in California through the *Medi-Cal* (Medicaid) program. DHCS maintains the State of California’s Medicaid Management Information System/Decision Support System (MMIS/DSS), an administrative database for claims paid to physicians and pharmacies. This database includes the number of prescriptions for ibuprofen and acetaminophen paid for by *Medi-Cal*. The database also includes paid doctors’ office visits for each child. We extracted both of these when creating our dataset. The data for this study were obtained from the MMIS/DSS for 2003 to 2010. Infants remain eligible for *Medi-Cal* for one year if they live with their mother. Thereafter, eligibility is determined annually. *Medi-Cal* also pays retroactively where eligibility is determined after initial hospital discharge. For a condition such as bronchiolitis, which typically presents beyond the neonatal period, and use of short-term medications, the combination of *Medi-Cal*’s characteristics and using age younger than one year as an inclusion criterion, limits the potential for misclassification of actually prevalent bronchiolitis cases or antipyretic use.

The database was mostly populated by manual coding of paper charts. This coding was performed by a variety of offices, clinics, billing companies, etc. Two diagnoses per visit are permitted.

### Study definitions and cohort inception

#### Cohort 1

Children had to be younger than 12 months of age and be diagnosed with their first episode of bronchiolitis or wheezing illness consistent with bronchiolitis to enter the cohort. They also had to have demonstrated prior or subsequent use of *Medi-Cal* as their payer for antipyretics.

#### Cohort 2

Children had to be younger than 12 months of age, be diagnosed with their first medically attended URI, not be diagnosed with prior or concurrent bronchiolitis or pneumonia, and fill a prescription for acetaminophen or ibuprofen or both. Patients were identified using the ICD-9 codes 460, 465.0, 465.8 and 465.9.

#### Cohort 3

We defined the inception visit as in Cohort 1, with the additional requirement that their caregiver had filled a prescription for acetaminophen or ibuprofen or both for the initial visit. Because Cohort 1 and 3 each required their own probability weighting models, this cohort is not fully nested in the cohort for Cohort 1.

### Outcomes

Our primary outcome was the number of visits to a health care provider for a wheezing illness consistent with bronchiolitis or asthma at 14, 30, and 365 days post inception into their cohort. Because parents may present more than one time for what is effectively the same illness, we created a secondary outcome of ‘episodes’ where an episode was defined as one or more visits within a seven-day period for our 12-month analyses. We also did this for the 14 and 30-day timepoints, but the results were almost identical to visit counts and we did not pursue them further. The diagnosis codes used are in Table 1. Sensitivity analyses comparing narrow and broader ICD-9-CM codes consistent with bronchiolitis were performed.

**Table 1:** Summary of ICD-9-CM codes included in our outcomes.

Bronchiolitis	Code
Acute bronchiolitis/bronchitis	466(*)
Acute bronchiolitis	466.1
Acute bronchiolitis due to RSV	466.11
Acute bronchiolitis due to other organism	466.19
Bronchitis	
Acute bronchitis	490
Bronchitis NOS	466.0
Asthma codes	
Asthma	493
Extrinsic asthma	493.0
Extrinsic asthma NOS	493.00
Extrinsic asthma with status asthmaticus	493.01
Extrinsic asthma with acute exacerbation	493.02
Intrinsic asthma	493.1
Intrinsic asthma NOS	493.10
Intrinsic asthma with status asthmaticus	493.11
Asthma NOS with acute exacerbation	493.12
Asthma NOS with (acute) exacerbation	493.9
Asthma NOS	493.90
Asthma with status asthmaticus	493.91
Asthma NOS with acute exacerbation	493.92
Bronchospasm or wheezing	
Acute bronchospasm	519.11
wheezing	786.07

Summary of ICD-9-CM codes used to define outcomes and entry criteria. NOS; not otherwise specified. Sensitivity analysis restricting outcomes to bronchiolitis or bronchitis (narrow diagnosis) did not change the results compared with including all diagnosis codes listed here (broad diagnosis). NOS; not otherwise specified.

(*) An outdated incomplete code found in the dataset.

### Exposure

Patients were classified as receiving acetaminophen if the caregiver filled a prescription for acetaminophen at the first visit; as receiving ibuprofen if the caregiver filled a prescription for ibuprofen at the first visit; and as receiving both if the caregiver filled prescriptions for both acetaminophen and ibuprofen at the first visit. Many infants were subsequently prescribed additional antipyretics over the following 12 months and we adjusted each analysis for these interval exposures. We performed robust negative binomial regression modelling the count of the number of visits and episodes at each time point. We obtained optimal model fit and specification, using Bayesian information criterion and measurement of link error respectively. We obtained optimal model fit and specification for Cohorts 1 and 3 using prescription counts for the 365-day follow-up and categorizing additional ibuprofen or acetaminophen use as any or none during the 14-day and 30-day follow-up periods. We obtained optimal model fit and specification for Cohort 2 when we categorized additional interval acetaminophen and ibuprofen at the median split, for the first 14 and 30 days of follow-up, and by tertile for the 12-month follow-up period for the analyses simulating an RCT during URI.

### Propensity score balancing

Propensity score analysis, subject to assumptions, allows simulation of randomization from observational data.[18, 19] On average, two subjects with the same propensity score will have the same potential outcomes and so comparing treatments between patients with the same propensity score will give an unbiased estimate of the effect of treatment.[20] Propensity scores are derived by constructing a model to predict individual subjects’ treatment assignment typically using probit or logistic regression models. The resulting inverse probability weights are used to simulate blocked randomized controlled trials (RCT).[21] Data management was performed using Stata 14 (Statacorp LLP, College Station, TX).

We derived a probit-based propensity score to estimate the probability that a child would be prescribed ibuprofen (either alone or with acetaminophen) or acetaminophen alone. We created variables for county level differences in ibuprofen prescription in those younger than two years and younger than six months. We also included age, previous diagnoses (e.g. gastrointestinal bleeding, renal impairment) which would make subsequent NSAID prescription less likely, and previous acetaminophen or ibuprofen use (which may make the prescriber more comfortable prescribing it again) in the propensity score model. We transformed independent variables and included interactions as necessary to ensure adequate model specification and fit.[22] A multinomial logit model was used to estimate the probability that an infant would have no prescription filled, a prescription filled for acetaminophen only, ibuprofen only, or both. The variables and interactions used in each model are shown in Table 2. We used the Stata package “mmws” to calculate the inverse proportional weights.[23] [24] For binary models, we derived inverse proportional weights from these scores using the Stata package “propensity”.[25] We restricted our sample to include only those subjects whose propensity scores fell within the range observed among those infants who did and did not receive ibuprofen.[25]

**Table 2:** Variables used in the propensity score models.

Multinomial logit model	Binary probit model	Binary probit model
Acetaminophen alone versus ibuprofen alone versus ibuprofen plus acetaminophen	Acetaminophen versus ibuprofen for URI	Acetaminophen versus ibuprofen for bronchiolitis
**Variable**	**Variable**	**Variable**
Year entered cohort	Year entered cohort	Year entered cohort
Age < 6 months at first prescription (binary)	Age < 6 months at first prescription (binary)	Age < 6 months at first prescription (binary)
Age	Age^2	Age^2
	Age^2 Interacted with age < 6 months at first prescription	Age^2 Interacted with age < 6 months at first prescription
	Male	Male
Prior medical history that could affect prescribing decision		
Any prior mild GI events	Any prior mild GI events	Any prior mild GI events
Any moderate or severe GI events	Any moderate or severe GI events	Any moderate or severe GI events
	Any prior renal impairment	Any prior renal impairment
Prior history of tolerating the drugs thereby affecting prescribing decision		
Previously had ibuprofen before cohort inception (binary)	Previously had ibuprofen (episodes categorized)	Previously had (episodes categorized)
Previously had acetaminophen (episodes categorized)	Previously had acetaminophen (episodes categorized)	Previously had acetaminophen (episodes categorized)
	Each of above interacted (one-way) with age < 6 months	Each of above interacted (one-way) with age < 6 months
Previously had acetaminophen (episodes categorized) ^2	Previously had ibuprofen (episodes categorized) ^2	Previously had acetaminophen (episodes categorized) ^2
Geographically prevalent prescribing practices		
Proportion of infants prescribed ibuprofen in that county	Proportion of infants prescribed ibuprofen in that county	Proportion of infants prescribed ibuprofen in that county
Proportion of infants < 6 months prescribed ibuprofen in that county	Proportion of infants < 6 months prescribed ibuprofen in that county	Proportion of infants < 6 months prescribed ibuprofen in that county
Seasonal and geographic variables		
County (Categorical)	County (Categorical)	County (Categorical)
Inception visit occurred during bronchiolitis season		Inception visit occurred during bronchiolitis season

Variables chosen based on perceived importance and contribution to model fit. URI; upper respiratory tract infection.

We used these inverse probability weights to adjust the robust negative binomial regression modeling the count of the number of visits and episodes at each time point. This process allowed us simulate a blocked RCT with enrollment occurring when an infant was first prescribed ibuprofen or acetaminophen for a URI.[21] The success of balancing was assessed using standardized differences between the pre-and post-balanced samples.

### Post-balancing analysis adjustment

The process of stratifying, matching, minimizing or otherwise blocking an RCT can introduce correlation among the treatment groups; some argue that this violation of independence requires adjusting the analysis for these covariates.[26] In this adjustment, these covariates were non-significant and had little effect on other variable coefficients. We modeled the interaction between treatment assignment and subsequent acetaminophen and ibuprofen prescription. The variables used for adjustment in the main analyses are listed in Table 3.

**Table 3:** Models and interactions in primary analyses.

URI model 365-day follow-up	URI model 30-day follow-up	URI model 14-day follow-up
Drug	Drug	Drug
Tertile of subsequent ibuprofen exposure	Subsequent ibuprofen exposure	Subsequent ibuprofen exposure
Tertile of subsequent acetaminophen exposure	Subsequent acetaminophen exposure	Subsequent acetaminophen exposure
Interactions of subsequent ibuprofen with acetaminophen use	Interactions between acetaminophen and ibuprofen	Interactions between acetaminophen and ibuprofen
Incepted during bronchiolitis season	Incepted during bronchiolitis season	Incepted during bronchiolitis season
	Month incepted	Month incepted
	Year interacted with bronchiolitis season	
Year enrolled	Year enrolled	Year enrolled
Age	Age	Age
Age < 6 months	Age < 6 months	Age < 6 months
**Bronchiolitis model 365-day follow-up**	**Bronchiolitis model 30-day follow-up**	**Bronchiolitis 14-day follow-up**
Drug	Drug	Drug
Number of prescriptions of ibuprofen during follow-up period	Ibuprofen during follow-up period (binary)	Ibuprofen during follow-up period (binary)
Number of prescriptions of acetaminophen during follow-up period	Acetaminophen during follow-up period (binary)	Acetaminophen during follow-up period (binary)
Interactions between acetaminophen and ibuprofen	Interactions between acetaminophen and ibuprofen	Interactions between acetaminophen and ibuprofen
Incepted during bronchiolitis season	Incepted during bronchiolitis season	Incepted during bronchiolitis season
Year enrolled	Year enrolled	Year enrolled
	Year interacted with bronchiolitis season	
Age	Age^2	Age^2
	Age less than six months	Age less than six months

Variables were chosen based on perceived importance and contribution to model fit. URI; upper respiratory tract infection.

### Follow-up period

We created follow-up time points at 14, 30, and 365 days after the inception visit. We censored follow-up at 365 days following the inception visit. If there was no evidence of a visit after each of the study follow-up time points, we censored the data at the last known visit in the first year of Medi-Cal eligibility or at 365 days if the child continued to use Medicaid services in his second year of life.

We restricted our primary analysis to include only infants whose inclusion in the study occurred up to and including2009. We did this because infants enrolled in 2010 could not by definition complete 365-day follow-up.

### Sensitivity analysis

#### Outcomes

Sensitivity analysis restricting outcomes to bronchiolitis or bronchitis (narrow diagnosis) did not change the results compared with including all diagnosis codes listed in Table 1 (broad diagnosis). We therefore report our outcomes using broad diagnosis codes.

#### Potential unmeasured exposure in Cohort 1

A particular concern for Cohort 1 was that parents who were not prescribed an antipyretic for their child would nonetheless administer one. (Although also possible for the other cohorts, the fact that all of these filled a prescription for at least one antipyretic makes this less a concern.) Lin et al has argued that in this situation investigators should examine how the findings of an observational study would be affected by variations in assumptions made about unmeasured confounders. If the conclusions are insensitive to a wide range of plausible assumptions, causal conclusions become more defensible.[27]

We therefore did a sensitivity analysis where we investigated the effect of a wide range of plausible scenarios where patients who did not fill a prescription for an antipyretic might nonetheless have received one. In this sensitivity analysis, we randomly assigned those who did not fill a prescription for any antipyretic as if they had in fact received acetaminophen, ibuprofen, both or neither, and repeated our analysis using the new drug assignments. We repeated this process 300 times and accepted the median point estimate and standard errors. We performed this sensitivity analysis to reassure us that the direction of effect in Cohort 1 was correct.

In secondary sensitivity analyses, we included children enrolled in 2010 and up to 24 months of age for the 14 and 30-day outcomes. In another sensitivity analysis, we restricted the definition of 365-day follow-up to those infants who used their Medi-Cal benefit 366 days or later after their inception into the cohort. We used seemingly unrelated estimation to test coefficients between drugs at each time-point and to test the coefficients in the final models between those infants with narrowly and broadly defined illness consistent with bronchiolitis based on ICD-9-CM diagnosis codes.

## Results

**Fig 1:**
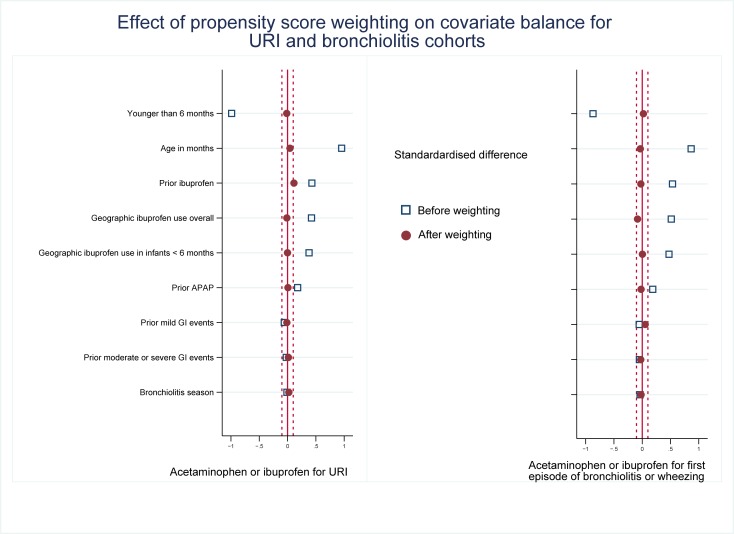
Effect of weighting scheme on covariate balance. The x-axis shows the standardized difference between treated and untreated subjects before and after adjustment.

Patient characteristics are shown in Table 4. The treatment groups were unbalanced at the inception visit in all three analyses. This imbalance was successfully corrected using propensity scoring Fig 1.

**Table 4:** Demographics of each cohort.

Variable		Cohort 1	Cohort 2	Cohort 3
	N	28,870	62,255	14,491
Male	n	16,086 (56%)	31,776 (51%)	7,630 (53%)
Age	Med	5.52	5.29	6.47
	IQR	3.44, 8.03	2.91, 8.00	4.69, 8.76
Age < 6 months	n	16,060 (56%)	35,534 (57%)	6,296 (43%)
Year	2003	1,996 (7%)	5,218 (8%)	1041 (7%)
	2004	4,417 (15%)	9,550 (15%)	2,256 (16%)
	2005	4,775 (17%)	10,747 (17%)	2,426 (17%)
	2006	4,418 (15%)	9,468 (15%)	2,152 (15%)
	2007	4,599 (16%)	9,226 (15%)	2,195 (15%)
	2008	4,330 (15%)	8,668 (14%)	2,168 (15%)
	2009	4,335 (15%)	9,358 (15%)	2,253 (16%)
Acetaminophen naïve	n	14,213 (49%)	34,602 (56%)	5,822 (40%)
Ibuprofen naïve	n	26,838 (93%)	59,002 (95%)	12,999 (90%)
Completed 14-day follow-up	n	27,130 (94%)	59,466 (96%)	14,059 (97%)
Completed 30-day follow-up	n	25,586 (89%)	55,879 (90%)	13,510 (93%)
Completed 365-day follow-up	n	18,550 (64%)	39,301 (63%)	10,536 (73%)

Demographics of each cohort. Characteristics of infants in each cohort. Data only fully available from 2004. Med; median, IQR; interquartile range.

### Cohort 1. Ibuprofen versus acetaminophen versus neither for bronchiolitis

We observed consistent large effects favoring acetaminophen or ibuprofen over no drug, adjusted incident rate ratio (aIRR) 0.18, (95% CI 0.15–0.22 at 365 days for acetaminophen), aIRR 0.18, (95% CI 0.12–0.27) at 365 days for ibuprofen, and ibuprofen plus acetaminophen aIRR 0.12, (95% CI 0.05–0.32) at 365 days. The results were similar when episodes rather than visits were counted. There was as similar reduction in episodes of wheezing at 30 and 14-day follow-up (Table 5). The apparently large effect size was markedly reduced in magnitude, but not direction in sensitivity analysis. (Table 5)

**Table 5:** Acetaminophen versus ibuprofen versus neither for bronchiolitis.

Drug prescribed		Cohort 1				Sensitivity Analysis				
						Median			Extreme	
	(days)	n	aIRR	95%lb	95% ub	aIRR	95% lb	95% ub	95% lb	95% ub
No antipyretic	365	9,863	1	-	-	1	-	-		
Acetaminophen only	365	7,011	0.18	0.15	0.22	0.48	0.39	0.57	0.39	0.59
Ibuprofen +/- acetaminophen	365	1,676	0.15	0.09	0.23	0.46	0.40	0.54	0.26	0.60
Ibuprofen only	365	1,454	0.18	0.12	0.27					
Ibuprofen + acetaminophen	365	222	0.12	0.05	0.32	0.35	0.28	0.44	0.25	0.49
Total		18,550								
No antipyretic	30	13,106	1	-	-	1	-			
Acetaminophen only	30	10,337	0.14	0.13	0.15	0.31	0.27	0.35	0.24	0.41
Ibuprofen +/- acetaminophen	30	2,143	0.14	0.13	0.14	0.36	0.28	0.45	0.25	0.53
Ibuprofen only	30	1,858	0.17	0.16	0.18					
Ibuprofen + acetaminophen	30	285	0.11	0.10	0.12	0.29	0.21	0.39	0.19	0.24
Total	30	25,586								
No antipyretic	14	13,844	1	-	-	1	-			
Acetaminophen only	14	11,044	0.13	0.12	0.15	0.29	0.25	0.34	0.22	0.41
Ibuprofen +/- acetaminophen	14	2,242	0.13	0.12	0.13	0.35	0.25	0.47	0.22	0.57
Ibuprofen only	14	1,943	0.15	0.14	0.16					
Ibuprofen + acetaminophen	14	299	0.10	0.10	0.11	0.28	0.20	0.39	0.17	0.47
Total	14	27,130								

Results for Cohort 1. Subsequent doctor visits for wheezing in infants prescribed antipyretics for a first episode of wheezing or bronchiolitis. In the sensitivity analysis we assumed that some children who were not prescribed ibuprofen or acetaminophen at their inception visit nonetheless were administered it at home. We randomly assigned a new treatment group i.e. no antipyretic/acetaminophen/ibuprofen or both acetaminophen and ibuprofen to children who were prescribed neither drug at their inception visit. Median refers to the median result under simulation. The extreme bounds refer to the lowest and highest 95% that occurred in the simulation. The direction but not the magnitude of the effect is preserved in sensitivity analysis. aIRR; adjusted incidence rate ratio, n number of patients, lb; lower bound for 95% confidence interval, ub; upper bound for 95% confidence interval.

### Cohort 2. Ibuprofen versus acetaminophen for URI

Ibuprofen alone or with acetaminophen was associated with a small reduction in subsequent visits for, and episodes of wheezing illness at one-year follow-up (aIRR 0.87, 95% CI 0.76–1.00). At 30-day follow-up, we observed more doctor visits for those receiving ibuprofen (aIRR 1.28 95% CI 12.04–1.58), particularly in combination with acetaminophen (aIRR 1.66, 95% CI 1.08–2.55), but not when ibuprofen alone was prescribed (Table 6). This was also true for episodes of wheezing (Table 7). We did not find any effect at 14-day follow-up. Sensitivity analysis including children up to 24 months for the 14 and 30-day outcomes showed broadly similar results to our primary analysis except that we did not find increased visits at 30-day follow-up in Cohort 2.

**Table 6:** Effect of antipyretics on visits for wheezing illness.

Drug		Upper respiratory tract infection Cohort 2				Bronchiolitis Cohort 3			
	Time point	n	aIRR	95% lb	95% ub	n	aIRR	95% lb	95% ub
No antipyretic	365	na	na	na	na	na	na	na	na
Acetaminophen only	365	30,805	1	-	-	7,748	1	-	-
Ibuprofen +/- acetaminophen	365	8,497	0.868	0.755	0.996	2,782	0.791	0.682	0.916
Ibuprofen only	365	6,924	0.832	0.715	0.968	2,450	0.818	0.702	0.953
Ibuprofen + acetaminophen	365	1,572	1.027	0.761	1.386	332	0.632	0.417	0.958
**Total**		39,301				10,536			
No antipyretic	30	na	na	na	na	na	na	na	na
Acetaminophen only	30	45,574	1	-	-	10,178	1	-	-
Ibuprofen +/- acetaminophen	30	10,305	1.283	1.039	1.583	3,332	1.026	0.779	1.352
Ibuprofen only	30	8,419	1.209	0.958	1.152	2,926	1.070	0.804	1.424
Ibuprofen + acetaminophen	30	1,886	1.662	1.081	2.554	406	0.794	0.353	1.784
**Total**	30	55,879				13,510			
No antipyretic	14	na	na	na	na	na	na	na	na
Acetaminophen only	14	48,709	1	-	-	10,615	1	-	-
Ibuprofen +/- acetaminophen	14	10,757	1.021	0.789	1.323	3,444	1.00	0.784	1.284
Ibuprofen only	14	8,807	0.966	0.723	1.291	3,022	0.984	0.772	1.255
Ibuprofen + acetaminophen	14	1,950	1.282	0.790	2.080	422	1.116	0.492	2.532
**Total**	14	59,466				14,059			

Adjusted incident rate ratio (aIRR) for subsequent wheezing following the initial infection with 95% upper (ub) and lower bound (lb) confidence intervals (CI). na, not applicable;—not calculated for referents; p, probability value. The variables used in adjusting the estimates are in Table 2.

**Table 7:** Effect of antipyretics on episodes of wheezing illness.

Acetaminophen versus Ibuprofen		Bronchiolitis Cohort 1				URI Cohort 2				Bronchiolitis Cohort 3Cohort 3			
	Days follow-up	n	aIRR	95% lb	95% ub	n	aIRR	95% lb	95% ub	n	aIRR	95% lb	95% ub
No antipyretic	365	9,863	1	-	-	na	na	na	na	na	na	na	na
Acetaminophen only	365	7,011	0.185	0.154	0.224	30,805	1	-	-	7,748	1	-	-
Ibuprofen +/-acetaminophen	365	1,676	0.140	0.130	0.150	8,496	0.883	0.774	1.019	2,783	0.768	0.660	0.893
Ibuprofen only	365	1,454	0.167	0.106	0.265	6,924	0.853	0.733	0.992	2,450	0.794	0.678	0.931
Ibuprofen + acetaminophen	365	222	0.114	0.046	0.282	1,572	1.005	0.781	1.414	332	0.613	0.411	0.913
**Total**		18,550				39,301				10,530			

This analysis examines episodes rather than visits. In this analysis, visits for wheezing illness within a 7-day period were counted as a single episode. Adjusted incident rate ratio (aIRR) for subsequent wheezing following the initial infection with 95% upper (ub) and lower bound (lb) confidence intervals. The variables used in adjusting the estimates are in Table 2. na, not applicable;—not calculated for referents; p, probability value; URI, upper respiratory tract infection.

### Cohort 3. Ibuprofen versus acetaminophen for bronchiolitis

Ibuprofen alone and ibuprofen plus acetaminophen were associated with decreased visits for, and episodes of, subsequent wheezing at 365-day (aIRR 0.79, 95% CI 0.68–0.92), but not earlier timepoints, when compared with acetaminophen. These results are detailed in Tables 6 and 7.

**Fig 2:**
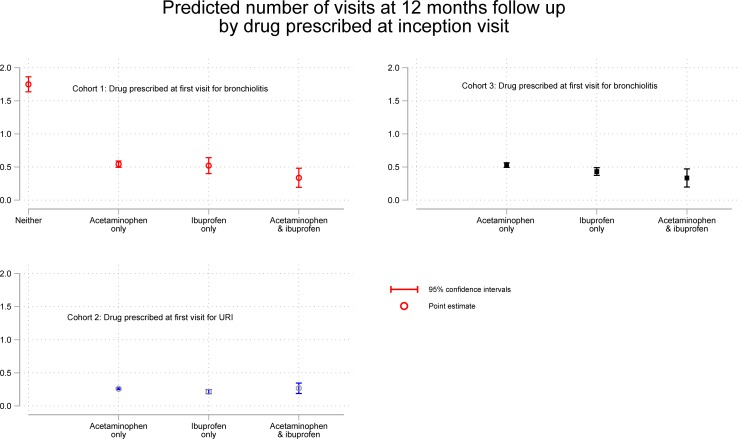
Effects of antipyretics on subsequent wheezing. The predicted number of visits with 95% confidence intervals by treatment at each time point for each analysis.

The results of all three analyses are shown in Fig 2.

### Sensitivity analysis

#### Cohort 1

In the sensitivity analysis where we randomly assigned a new treatment group (i.e. no antipyretic or acetaminophen alone or ibuprofen alone or both acetaminophen and ibuprofen to children who were prescribed neither drug at their inception visit) we observed generally decreased magnitude but preserved direction of effect favoring the administration of antipyretics to these children. The possible range of effect sizes for each drug at each time point is shown graphically in the S1 Appendix.

In post-hoc tests of the additional sensitivity analyses comparing coefficients across time points, we observed significant differences between the coefficients for acetaminophen and ibuprofen (*p*<0.001) between 365, 30, and 14 days follow-up but not between the coefficients for ibuprofen and the combination of ibuprofen with acetaminophen. The effect sizes were significantly larger between infants with narrowly rather than broadly defined bronchiolitis at cohort inception except for the combined ibuprofen plus acetaminophen group.

#### Cohort 2

Sensitivity analysis across all time points tested simultaneously and in pairs demonstrated significant differences between ibuprofen between 365-day, 30-day, and 14-day follow-up and comparing coefficients for combined acetaminophen and ibuprofen between 14-day and 30-day follow-up (p = 0.0007–0.0178). Although generally similar to the primary findings, our sensitivity analysis, which included children up to 24 months of age, failed to find increased wheezing at 30 days in the ibuprofen group.

#### Cohort 3

Sensitivity analysis across time points showed incremental benefit between follow-up days 365, 30, and 14, but not between day-14 and day 30-follow-up in the ibuprofen group. The effect sizes were not significantly larger between infants with narrowly rather than broadly defined bronchiolitis at cohort inception. Overall, these sensitivity analyses support the direction and magnitude of our findings.

## Discussion

We found modestly decreased subsequent doctor visits for, and episodes of, wheezing illness when ibuprofen rather than acetaminophen was prescribed at the first episode of bronchiolitis. The combination of both antipyretics was associated with a larger effect size.

Initiating treatment with ibuprofen rather than acetaminophen at the URI stage was associated with a smaller effect size at one year. We observed a statistically significant increase in doctor visits at 30 days in the acetaminophen, and acetaminophen plus ibuprofen groups. This increase was not observed in our sensitivity analysis which included 75,578 patients up to 24 months of age. Our other findings remained consistent between the primary and sensitivity analyses.

Our results are complement bovine data showing that ibuprofen may modulate the host immune response to RSV that may decrease the later formation of IgE to bystander antigens.[7] NSAIDs are a routine of part of treatment of bovine respiratory complex which typically is triggered by bovine RSV.[28] Veterinary research shows also decreased subsequent respiratory illness when ketoprofen, a NSAID, is added to the drinking water of piglets.[29] However NSAIDs also increase the peak and duration of viral shedding during the initial acute illness in humans and animals.[7, 17]

Cohort 1, any antipyretic decreased subsequent wheezing at every time point, is the most provocative and least certain of our findings. It is the most provocative because it suggests a substantial long-term benefit to prescribing antipyretics for a first episode of bronchiolitis and is consistent with observations from animal and vaccine studies. It is the least certain because the assumption of ignorability is much shakier than for the other cohorts. Our sensitivity analysis where we assumed that some infants who were not prescribed antipyretics nonetheless received them provides some reassurance. This sensitivity analysis showed a smaller more conservative but clinically important effect size. Most importantly the direction of effect was unchanged. Cohort 1 nonetheless has two competing interpretations. The first is that a first episode of wheezing not accompanied by fever differs from one accompanied by fever and is inherently more likely to recur. The second interpretation is that either ibuprofen or acetaminophen treatment of an initial episode of bronchiolitis or bronchospasm decreases subsequent wheezing, and that using both together may be more effective than either alone Definitively resolving these competing interpretations will likely require an RCT.

Our results for Cohort 2 contrast with those of Lesko et al that found decreased wheezing in febrile children treated with ibuprofen rather than acetaminophen at the 30 days. However, Lesko et al included those with a history of recurrent bronchospasm and excluded those younger than 6 months of age.[11] Since we included only those without prior wheezing, finding a difference at 1 year rather than 1 month is perhaps to be expected as bystander antibody formation in response to RSV infection takes time. Most URIs are not destined to become bronchiolitis. This dilutes the potential benefits of acetaminophen or ibuprofen while presumably increasing viral shedding and potentially increasing the duration of illness and infectivity of the child.

Cohort 3 had a progressively increasing benefit with ibuprofen and ibuprofen combined with acetaminophen. Although broadly speaking, ibuprofen acts peripherally and acetaminophen centrally, there is some overlap. Both drugs decrease fever and we speculate that decreasing fever may be associated with decreased antibody production to bystander antigens and therefore less subsequent wheezing.

### Limitations

There are limitations to our methodology.

#### Data quality

Administrative data from the MMIS/DSS database is of variable quality and does not include important covariates such as family history, parental smoking, breast feeding, daycare, sibling daycare, etc. We used pharmacy payment data as a proxy for drug use, but we cannot know how much, and for how long, parents administered it. Despite propensity based inverse proportional weighting’s promise of reaching causality from observational data, ignorability is not assured for Cohort 1 and full adjustment of other covariates in the regression of outcomes was not possible in Cohorts 2 and 3. Our work demonstrates association but does not prove causation.

#### Follow-up

We had successful follow-up in between 64% and 73% at one year. Successful follow-up required both successful re-enrollment in Medicaid and Medicaid payment for a doctor visit at or after 12 months of age. During the period covered by the study, California Medicaid annual re-enrollment was not automatic, and disenrollment occurred on the child’s first birthday if the parent did not actively seek to renew coverage. Typical Medicaid disenrollment rates for this payment were 27% at end of the first year of life.[30] This includes families whose eligibility is disrupted by marriage or changes in financial circumstances, those who obtain other insurance, and those who feel the marginal benefits are outweighed by the costs of reapplying.[30] In California, this last group can re-enroll and obtain retrospective coverage for hospitalizations and emergency room visits should the need arise. Finally, a myriad of administrative and software problems can also lead to mistaken failure to re-enroll or even disenrollment. Our study cannot address any of these.

#### Adjustment variables

Subsequent prescriptions for antipyretics may have value as an indicator of a child’s tendency to acquire and parental tendency to seek medical advice for febrile illnesses in addition to its role in ongoing immunomodulation. Adjusting for these subsequent antipyretic prescriptions improved model fit although non-adjustment made little difference to the coefficients of interest.

We assumed that parents who obtained their antipyretics at no cost to themselves, would not buy additional supplies over the counter (OTC). This is a more realistic assumption than it appears. By law, there is no monetary barrier to parents obtaining or filling a prescription, and in our experience, parental resistance to being directed to purchase antipyretics OTC is high. Even nominal co-pays of $0.50-$3.00 are a substantial hurdle to Medicaid recipients filling prescriptions.[31] Prescriptions are frequently telephoned to pharmacies by office staff, and in the US, ibuprofen purchased OTC carries a warning not to administer it to infants younger than six months of age. Some children may have received unrecorded left-over medications from siblings, although it is unclear that this would differ by treatment group.

#### Attempting to ascertain causality from observational data

The promise of using propensity scored inverse proportional weighting method is the ability to mimic RCT design and potentially extract causality from observational data. Even where assumptions of ignorability were met, the promise may fall short because we can only adjust for recorded variables.

Cohort 1 falls short in this respect. However, the primary purpose of Cohort 1 is to ascertain the directionality of any effect. Taken with our sensitivity analysis, Cohort 1 does provide reassurance regarding the likely direction of effect of antipyretics in bronchiolitis. This matters because future research aims to improve the outcome of infants with bronchiolitis. Prior to undertaking RCTs, we need to know that antipyretics likely decrease subsequent wheezing following bronchiolitis. Otherwise, we risk designing RCTs that determine only whether ibuprofen has a greater or lesser effect than acetaminophen without knowing if we should be using either. Our results support a trial that randomizes infants in their first episode of bronchiolitis but probably not URI to receive antipyretics or placebo even in the absence of fever. This RCT will need careful planning to manage fever that develops after randomization; but the effect sizes we found are sufficient to make such an RCT feasible.

## Conclusions

In conclusion, children who are prescribed antipyretics for a first episode of bronchiolitis may have less subsequent wheezing than those who are not. We found fewer episodes of subsequent wheezing for those prescribed ibuprofen, and ibuprofen combined with acetaminophen, compared with acetaminophen alone. There was a smaller effect for antipyretic treatment of a first URI at 365-day, but not earlier follow-up.

## Supporting information

S1 Appendix Range of possible coefficients for each drug using various sensitivity analyses. (PDF)
