# Supplementary material for: Wheezing after the use of acetaminophen and or ibuprofen for first episode of bronchiolitis or respiratory tract infection
Source: PLoS One. 2018 Sep 13;13(9):e0203770. doi: 10.1371/journal.pone.0203770 (PMC6136746; doi:10.1371/journal.pone.0203770)
Supplement: S1 Appendix — (PDF) [file pone.0203770.s001.pdf]

S1 Appendix

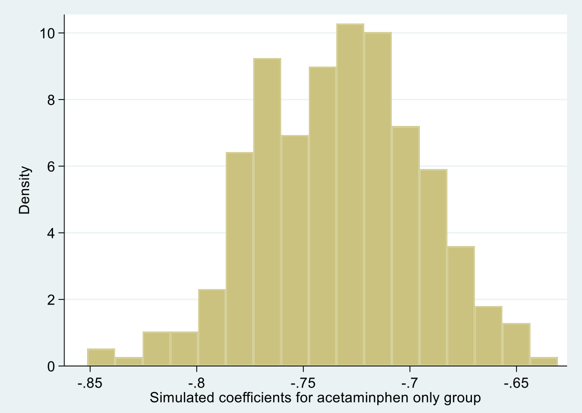

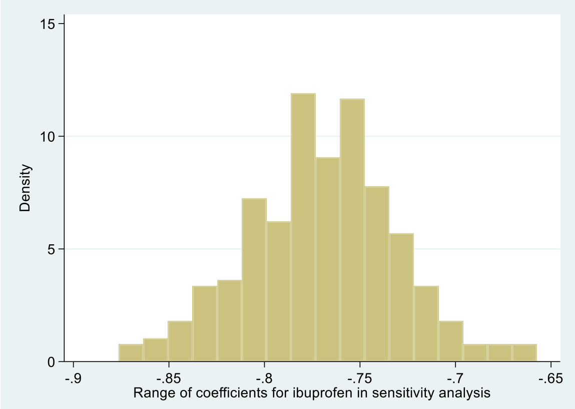

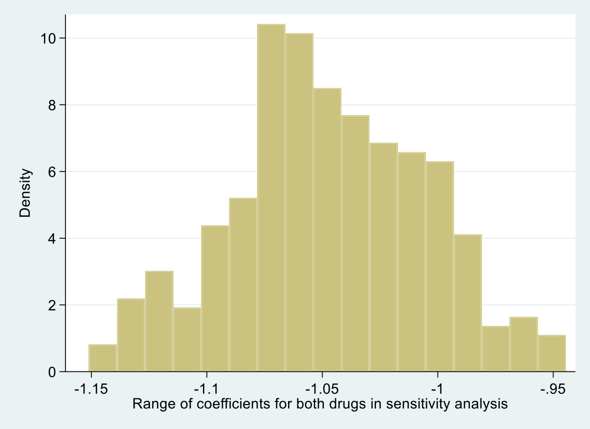

These graphs show the range of possible coefficients for each drug group in 300 simulations. This is for the 1 year follow up

In the sensitivity analysis we assumed that some children who were not prescribed ibuprofen or acetaminophen at their inception visit nonetheless were administered it at home. We randomly assigned a new treatment group i.e. none/acetaminophen/ibuprofen or both drugs to children who were prescribed neither drug at their inception visit.

The sensitivity analysis coefficients continue to show a benefit if effect favoring antipyretics although the effect is much more modest. We now report the median of these alongside the original estimates in the main manuscript.

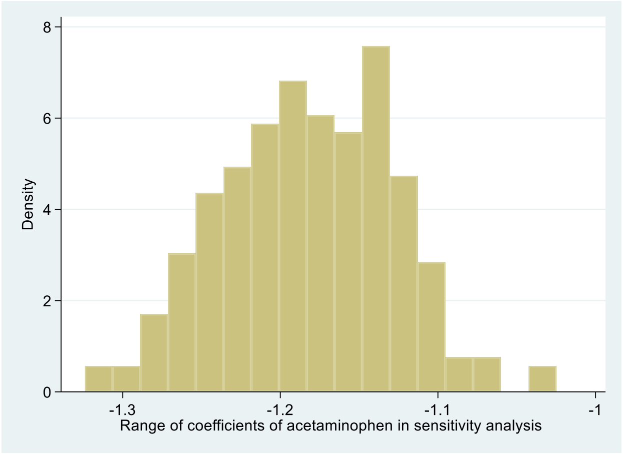

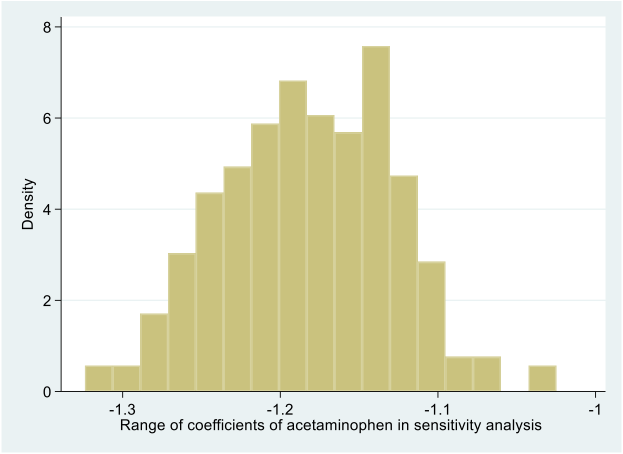

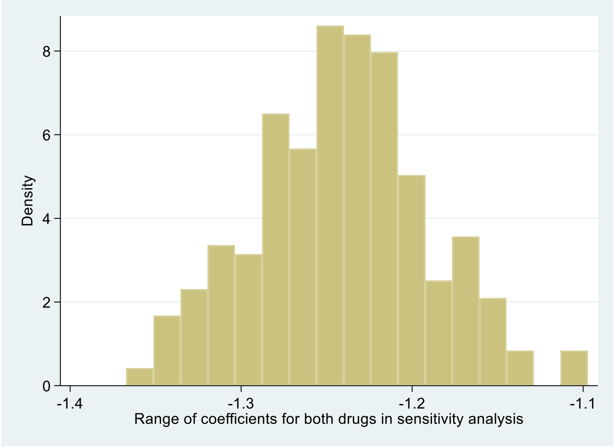

These graphs show the range of possible coefficients for each drug group in 300 simulations. This is for the 30-day follow up

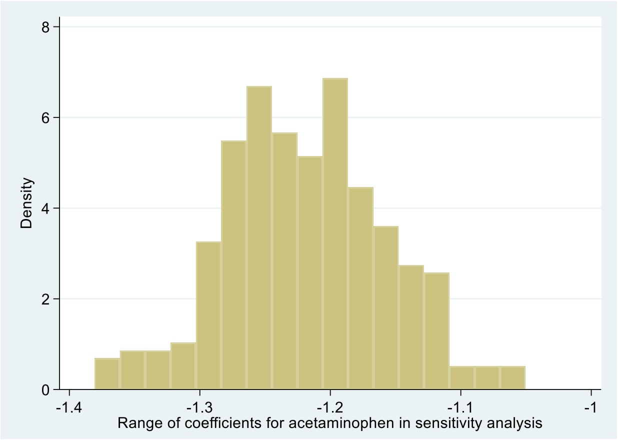

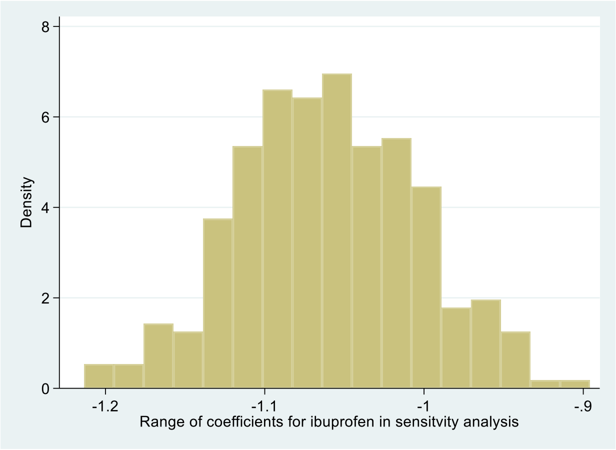

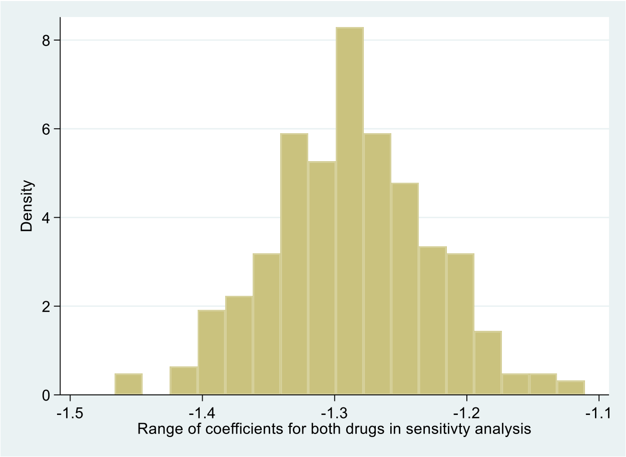

These graphs show the range of possible coefficients for each drug group in 300 simulations. This is for the 14-day follow up

Alternative models

Effect of not adjusting for subsequent antipyretic use in selected models

Doctor visits for subsequent wheezing at 14-day follow up

Adjusted for subsequent antipyretics					Not adjusted for subsequent antipyretics		
Drug group	aIRR	95%lb	95%ub		aIRR	95%lb	95%ub
APAP only	1	-	-	-	1	-	-
Ibuprofen +/- APAP	1.021	0.789	1.323	0.872	1.042	0.800	1.358
Ibuprofen only	0.966	0.723	1.291	0.817	0.974	0.729	1.302
Ibuprofen + APAP	1.282	0.790	2.080	0.315	1.377	0.779	2.435

Adjusted for subsequent antipyretics				Not adjusted for subsequent antipyretics		
Drug group	aIRR	95%lb	95%ub	aIRR	95%lb	95%ub
APAP only	1	-	-	1	-	-
Ibuprofen +/- APAP	1.283	1.039	1.583	0.949	0.779	1.156
Ibuprofen only	1.209	0.958	1.152	0.916	0.735	1.141
Ibuprofen + APAP	1.662	1.081	2.554	1.110	0.741	1.662

Doctor visits for subsequent (episodes of) wheezing at 365-day follow up

Adjusted for subsequent antipyretics				Not adjusted for subsequent antipyretics		
	aIRR	95% lb	95% ub	aIRR	95% lb	95% ub
APAP only	1	-	-	1	-	-
Ibuprofen +/- APAP	0.883	0.774	1.019	0.828	0.718	0.951
Ibuprofen only	0.853	0.733	0.992	0.864	0.761	0.982
Ibuprofen + APAP	1.005	0.781	1.414	0.999	0.913	1.095

All other adjustment as per models in manuscript.
